# Tribute to Mark Wainberg

**DOI:** 10.1186/s12977-017-0361-6

**Published:** 2017-06-28

**Authors:** Eric J. Arts, Anne Gatignol, Andrew J. Mouland, Chen Liang, Matthias Götte, Hugo Soudeyns

**Affiliations:** 1 Department of Microbiology and Immunology, Western University, London, Canada; 2 McGill AIDS Centre, Lady Davis Institute, McGill University, Montreal, Canada; 3 Department of Microbiology and Immunology, University of Alberta, Edmonton, Canada; 4 Département de microbiologie, infectiologie et immunologie, Université de Montréal, Montreal, Canada

## Introduction/preface

How do you remember Dr. Mark Wainberg? That is the question we posed to his trainees, former students, post-doctoral fellows and colleagues. Listed below are just a sample of the overwhelming response to this question we received from around the world. Together, Hugo Soudeyns, Anne Gatignol, Andrew Mouland, Matthias Götte, and Chen Liang compiled these tributes and I penned this introduction on behalf of my colleagues.
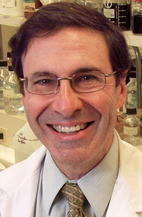


Over the past couple of weeks since his passing, I have been traveling in Africa and Europe. So many of our colleagues have expressed their condolences to me but more revealing was the stories they shared about the direct impact Mark had on their professional and personal lives. In a few instances, some of the leaders in our research field, well established professors and physicians, told me that if it wasn’t for Mark’s sound advice, they would not have reached their career goals. Now, I suspect that these individuals would have still achieved success and contributed amazing discoveries to the HIV/AIDS field but Mark just had an uncanny sense of how to be supportive and provide constructive advice. I remember feeling sorry for myself when I failed in getting my first NIH grant after joining the faculty. Aside from having my ego bruised by realistic criticisms of my research grant, I was also staring at that ominous tenure clock at a private, prestigious university where I suddenly felt out of place. I called Mark sounding defeated and depressed but Mark was not supportive of my sulking. In his slightly angry but nurturing tone (a trademark of Mark’s), he simply let me know that “Eric, success in science is not related to intelligence or smart ideas. You need to move your research forward, publish and most importantly, persevere. If you can handle that, the grants will come and the research field will benefit.” For the last 20 years, this has remained my mantra in research and one that I happily share with my trainees. Finally, Mark was deeply religious but as my colleague, Jonathan Schapiro wrote, “unlike many who use their religion as an excuse for racism or violence”, Mark was welcoming to all and his lab was vibrant mosaic with all ethnicities, races, religions, and partner preferences. Most important, he was well acquainted with the community in Canada living with HIV/AIDS and was their advocate regardless of lifestyle.

## Research contributions

As you will read below, most of his colleagues and former trainees wanted share personal memories of Mark intertwined with his research accomplishments and impact on HIV/AIDS treatment and care. Many of us forget Dr. Wainberg’s many contribution to research because Mark did not live in the past and was always looking forward to a new drug, a new resistance pathway, and new possibilities for treatment. Many of us including myself fall victim of framing a question at a conference by discussing “our” past publication or data. Mark was never going to lecture you on his contribution to AZT resistance [1–3], discovery of 3TC inhibition and resistance [4–6], characterization of early events of reverse transcription [7, 8], description of early Vpu activity and CD4 downmodulation [9–12], the low fidelity/fitness of 3TC resistant virus [13–18], or description of alternative drug resistance pathways in non-subtype B HIV [19–21] (50+ articles).
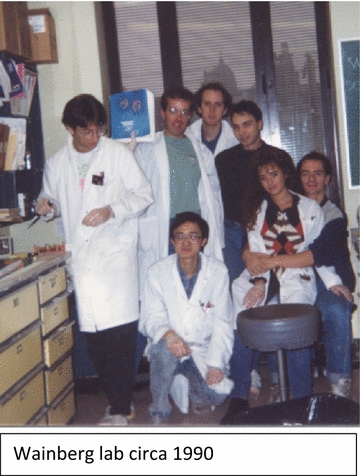


Our research field has had many powerhouse researchers in HIV drug resistance but no one had contributed more or had remained an integral part and leader for over 30 years. Search “Wainberg MA” and “HIV resistance” in Pubmed and you will get a list of nearly 300 articles (of his total 700+) that involved Mark’s direction or contribution. His most recent contribution was resistance to Dolutegravir (DTG). Mark predicted that DTG would be a successful antiretroviral drug for combination therapy based on his in vitro studies showing the slow emergence and high fitness cost of DTG resistance [22–27]. The Viking [28] and Spring [29, 30] clinical studies largely proved him correct but despite his 44 publications on HIV resistance to DTG, there remains doubt on the impact of these fitness costs. Mark reveled in these controversial and heated discussions. He pushed our boundaries like announcing the DTG may be effective in monotherapy [31] which was meant to be provocative and prevent our complacency in treatment and development of new drugs.

Mark was also passionate about getting the best treatment options for Africa [32, 33] and was highly critical of the use of NNRTI in first line treatment [34]. He was concerned about rapid emergence and transmission of NNRTI in low-to-middle income countries (LIMCs) which is now a public health crisis. Mark always argued that we as a research community have not done enough to avert this crisis despite our hundreds of publications forecasting the concerns of NNRTI resistance in LMICs Mark’s 62 publications on NNRTI resistance.

Mark could draw a crowd and then make bold, provocative, and premeditated statements to re-direct or re-focus the HIV/AIDS research field to address very important, life-saving treatment or prevention approaches. Who else remembers Mark asking an audience at the International AIDS Conference in 1989, “Has anyone else observed AZT resistance in treated patients?” Soon after Brendan Larder [35] then Mark [1] reported the first evidence of HIV resistance to antiretroviral drugs these clinical observations started our somber efforts to find better drugs and combination treatments.

Who remembers the publication by Mark and Vinayaka Prasad describing the poor fidelity and fitness of HIV carrying the 3TC resistant virus [36] that sparked a long and heated debate with John Coffin [37, 38]? Regardless of this controversy, most physicians still prefer to maintain a highly ARV experienced patient on Lamivudine or Emtricitabine despite the the appearance of the M184V resistance mutation especially if limited treatment options are available. Incidentally, Mark recently lost a bet to John Coffin based on another argument about drug resistance. Mark who never welched out on a bet and never parted with fine Scotch whiskey, did find a way out of this losing wager.

Who recalls Mark standing up at conferences (circa 2002/2003) and stating that we needed to start providing uninfected men (who have sex with men) with ddI as to prevent HIV infection [39, 40]? His logic was based on the limited use of Didanosine in treatment as well as the high genetic barrier to ddI resistance. Of course he did not expect this healthy population to take a foul tasting, horse pill that was largely abandoned from treatment but his provocative statement was meant to encourage Pharma to test their more accepted drugs for pre-exposure prophylaxis (PrEP). You may question Mark’s role in PrEP but you cannot question that Truvada has protected thousands from HIV infection.
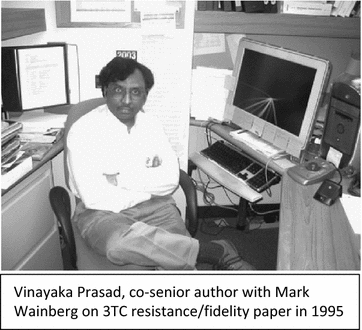


## Editor-in-Chief

Mark Wainberg is a name you could find on editorial boards of nearly every HIV or virology journal at some point in their publication history. He worked closely with Dr. Kuan-Teh Jeang on starting a couple of new journals but I don’t think Teh (or Mark) anticipated the impact that the Retrovirology journal would have on the field. Andrew Lever along with Mark served as co-editor-in-chief of Retrovirology following Teh’s passing and I am certain that Andrew will continue the excellent mandate of this journal. In addition to his role in *Retrovirology*, Mark was especially proud of starting the *Journal of the International AIDS Society* shortly after his term as President of IAS. Mark wanted a journal which featured both AIDS research in LIMCs as well as the research authored by LIMC researchers. This journal is shining example of proving to all of us that high impact, ground breaking HIV/AIDS research can and should originate in Africa, Southeast Asia, and other LIMCs around the world.

## Legacy in training

Dr. Mark A. Wainberg will always be remembered by his hundreds of trainees that came through his laboratory as undergraduate and graduate students, post-doctoral fellows, MD fellows, visiting scientist, technicians, and administrative support. He didn’t care about your title, race, ethnicity, gender, sexual preference or religion. He was going to take the time to talk to you, complain about something he didn’t like, or just ask you how you were doing. Long after you, left the lab, Mark continued to provide guidance and to ensure your continued success regardless of your career direction. I could provide a long list of past trainees that are now Professors, Department Chairs, and Vice Presidents in Industry. Almost none of us graduated with Science, Nature, or Cell papers but all of us contributed important research that was published in the work horse journals of our fields, were well cited, and stood the test of time. All left the Wainberg laboratory with an incredible knowledge base, outstanding presentation skills, and with the hard work ethic necessary to overcome the obstacles of future research endeavors.
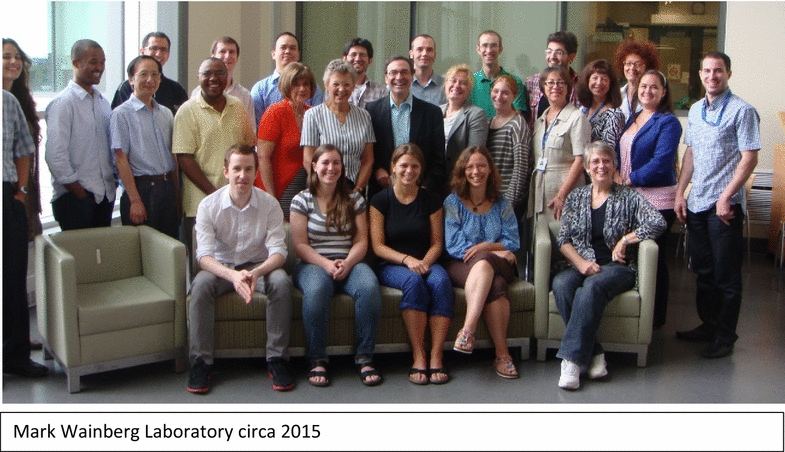


Of course, Mark was not perfect. His temper was legendary and yet, this was all fodder for endless stories and laughter that will be shared for decades to come. Just a few months after starting in the Wainberg lab in 1990, I remember being in his office and after he had just got a new phone. Some administrator had called and apparently shared some information that really soured his mood. Mark yelled into the phone and then tried to hang up. Getting tangled in the phone cord, the phone “somehow” got thrown across the room. He then walked across the office, pick up the mangled phone, gave me the typical Wainberg grin, and then told the administrator that his new phone stopped working and that he would need a new one. On occasion, Mark would lose his temper and inexplicably start yelling around the lab. His normal talking voice was typically 10 decibels above Mick Jagger's in concert so one can only imagine his yell. We would all cower and avoid eye contact but his anger was never really directed at anyone in particular. Without exception, following these rare fits of stress release, Mark would walk through the lab and apologize to everyone personally.
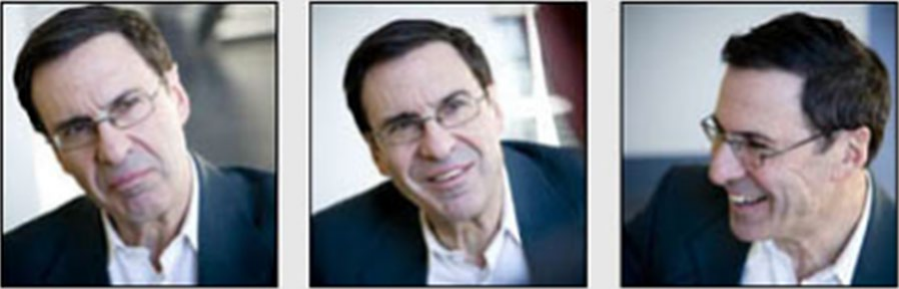


## Tributes

**Susan Moir:** Tenure-track investigator, NIH/NIAID

Years of contact with Mark: 20 years

“As an expat from Québec, I only saw Mark on occasion, mostly at conferences. In the winter of 2012 Mark invited me to give a talk at the annual Journées québécoises VIH. Of course, I agreed but asked if I could speak in English (French is the first language I learned but almost never use it in science and rusty from 20 years living in the US). He said no! That I needed to show respect for my French Canadian roots. Coming from Mark, an English-speaking Quebecer from the Jewish side of Montreal, I was so shocked by his refusal and by his insistence on speaking French, but pleasantly so! I cherish my roots, both French and English! I obliged and got through my talk in French, with the help of friends and colleagues sitting in the front row whispering translations as I needed them. I will never forget…je me souviendrai toujours! May you rest in peace, Mark.”

**Lisa Ross:** Senior Scientific Investigator ViiV Healthcare

Year of contact with Mark: 1998–2017

“After meeting Mark at a scientific conference almost 20 years ago, we discovered that we shared an interest in understanding how mutations in HIV impact drug susceptibility and understanding viral transmission of drug resistance. We continued our correspondence and conference interactions and he was always willing to offer suggestions or insights, yet also open to listening to the ideas of others. He actively mentored the careers of his students and was a passionate and respectful advocate for people living with HIV/AIDS. Besides being an insightful and thoughtful scientist, Mark had a keen sense of humor and a love of storytelling. One recent example occurred when his crown came off a tooth at the Sunday start of the 2016 HIV Glasgow meeting. As he wandered through Glasgow looking for an open dental clinic, a passerby asked if he needed assistance. Mark was delighted to later inform his conference colleagues that his good Samaritan turned out to be a dentist, who opened his office and fixed his tooth at no charge. He had an ability to frame his experiences into uplifting commentaries on the basic goodness of humanity. His joy and passion for life was truly infectious, and he will be sorely missed.” 
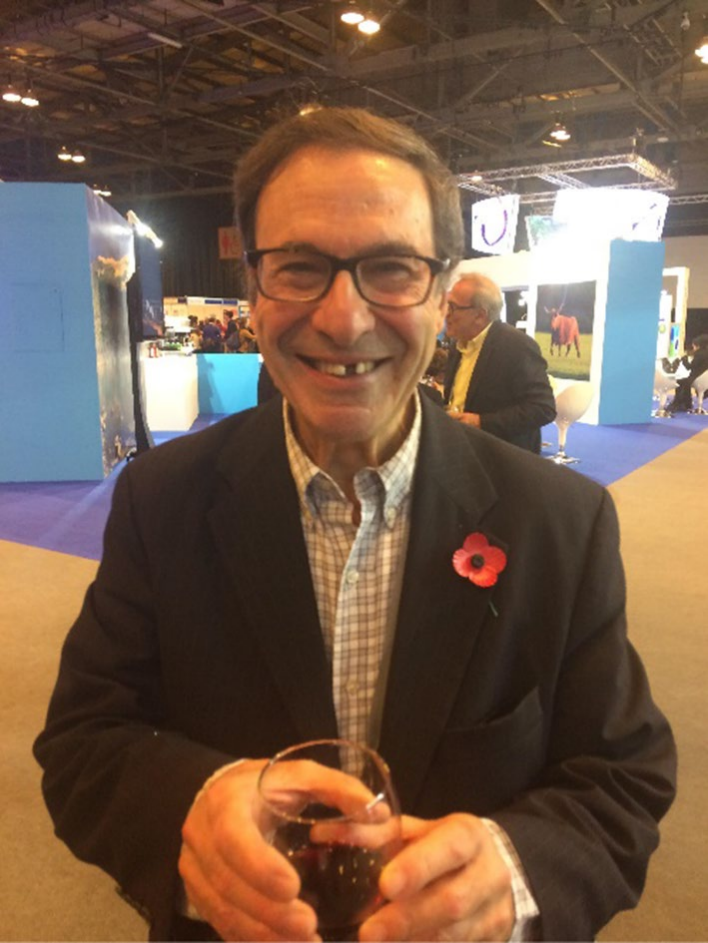


**Stefano Rusconi:** Associate professor in Infectious Diseases, DIBIC Luigi Sacco, University of Milan

Years of contact with Mark: 199–present

“Although I’ve never worked “for” Mark, I’ve known him since 1993, when I was a research fellow in Marty Hirsch’s lab. As well, I’ve never formally collaborated with him but I’ve met him at every conference and whenever he come to Milan. We discussed about research and he was always full of advice and his friendship was palpable, even because for many years he was among the few who could remember my name. Mark’s vision of life and science was enormous and very, very deep. Together with all these features, he always showed distinctive wit. I’ll miss him greatly both as a great man and as a great scientist.”

**Mark Mascolini:** Freelance writer/reporter

Year of contact with Mark: 1996–2016

“Mark approached me at an HIV meeting decades ago to say he liked my reporting. I felt enormously flattered but quickly learned flattery had nothing to do with his interest in my work. He peppered me with questions about how I covered HIV conferences. How did I review a meeting program? How did I decide what to report? How did I organize my articles? Would I read reviews he wrote and give my honest opinion? In about 2 minutes, Mark made me feel not like a grandstand observer of the HIV research parade, but like a respected colleague whose insights mattered. I’ll miss Mark a lot.”

**Vincent Calvez:** Professor of Medicine, Head of ANRS Virology Pitié-Salpêtrière Hospital

Years of contact with Mark: 1995–2017

“Mark was a great person: a model for us. He was a bright scientist and was also full of humanism. He was a friend of French scientists and a friend of France. He spent also plenty of time to train scientists from South African and Asian nations with seriousness and kindness. We miss him and we will never forget him.” Pr Vincent Calvez, Pr Diane Descamps and Pr Anne-Genevieve Marcelin (on behalf of the French Virology Network Group)”

**David Margolis:** Professor of Medicine, Director UNC HIV Cure Center, UNC Chapel Hill

Years of contact with Mark: 22 years

“Mark was a scientific external advisor for our research group 2011–2017 I knew and interacted professionally with Mark initially around our shared experience of working (at different times) in Bob Gallo’s group. This led to many stimulating years at meetings and conferences due to our overlapping work on HIV antiviral therapy, HIV drug resistance and later, on HIV persistence and the emerging effort in HIV cure research. Mark was always inspirational, energetic, and enthusiastic about everything he was doing and involved in. He generously gave his time and ideas to our cure research group as an external scientific advisor. Unlike many scientists, he was passionately and equally dedicated not just to the pursuit of new knowledge, but to the pursuit of the use and implementation of these discoveries. However, notwithstanding the strength of his intellect and force of his character, Mark was fun to be around, and never took himself too seriously. He is already greatly missed.”

**Julie Overbaugh**: Member and Associate Director Fred Hutchinson Cancer Research Centre

Years of contact with Mark: 1990s to present

“I met Mark at a gathering to organize a HIV meeting in the 1990s in Switzerland when I was still a relatively junior faculty member. I remember sharing a Gondola ride with Mark to our working dinner and then spending time with him at the dinner. I just recall him having lots of fun - maybe the wine helped a bit, as we were all enjoying the wine that night. It was nice to see someone who could work hard but also enjoy himself with colleagues. I am sorry that we have lost someone who was so passionate about his work and who stood up for things he believed in.”

**Nelson Michael:** Director, Military HIV Research Program, Walter Reed Army Inst of Research

Years of contact with Mark: 1990-present

“Mark was a standout scientist as well grounded in basic virology as he was in global health with a particular focus to bring HIV drugs to where they were needed most….in Africa. Colleagues abound with one of those attributes. To possess both, and do both well, made Mark a global treasure. He will be missed.”

**Pedro Cahn:** Scientific Director, Fundacion Huesped

Years of contact with Mark: 1997–2017

“Mark was a brilliant scientist and a great human being. Other colleagues will describe his outstanding scientific contributions. I just want to highlight his role as a human rights’ activist, and as such, one the founding fathers of ARV rollout in Africa. He was the one to nominate me as President of the IAS, not because of my merits, but because he was convinced that it was time to have somebody from a Southern country to be President. He nominated himself as my campaign Chief. He always greeted me saying. “Hola mi presidente”, even after my tenure. Today I say “Hasta siempre mi maestro”. The world was a better place with Mark.”

**Stephan Bour:** Vice President for Scientific Innovations, Digital Infuzion, Inc.

Years of contact with Mark: 1989 to present

“One of my most memorable moments is when, after a year in the lab, Mark asked me if I had enough data to write a paper. I told him I don’t think so, I don’t have much. He told me to bring him all my notebooks and results and 2 hours later, he had the outlines of 3 papers! Mark not only taught me how to be a scientist but also how to organize and communicate my insights to others and to the scientific community. That, as we all know, is in equal measure of importance as the science itself. I also treasure a few images and moments from my time in Mark’s lab. There was the blaring volume of his phone on speaker, the crazy late nights with the fantastic group of bright students he had assembled, and the day when on a dare I had his initials carved in my hair! Yes, that is the kind of admiration we all have in him. I can’t put into words how much I will miss him knowing that he is no longer in this world with us.”

**Aaron Donahue:** Institut Pasteur

Years of contact with Mark: 2008–2013

“One memory that reminds me of Mark’s enjoyment of science and his sense of humour is the HIV latency conference in St Marten. Mark was in the pool during a break between conference sessions, and got locked out of his hotel room when his key stopped working. The front desk couldn’t immediately replace it, and Mark, not wanting to miss the next talk, simply went into the conference in his wet bathing suit and a hotel bathrobe (with, of course, a big grin). After the session, to compensate Mark for the inconvenience, the hotel staff offered him a bottle of wine. He opened the wine that evening and shared it with a bunch of people. That same evening, we received the reviews on a manuscript that we had submitted. Not wanting to waste any time, we finished our wine and went to Mark’s room to send a response regarding the article, before returning to socialize for the rest of the evening.”

**Eugene Asahchop**: Postdoctoral Fellow, University of Alberta

Years of contact with Mark: April 2007–Dec 2012

“Dr. Mark Wainberg was my Ph.D. co-supervisor together with Dr. Cecile Tremblay. The majority of my Ph.D. research work was done in Mark’s lab. I worked in Mark’s lab from April 2007 as a research technician, and in September 2008 I enrolled for a Ph.D. in Microbiology and immunology at University of Montreal and graduated in December 2012.

As graduate students, Mark taught us the principle of a successful scientist. I will quote him “ As a scientist you must publish, if you don’t publish you perish”. After my Ph.D. defense during a farewell party for me, Mark made a statement during his speech and I will quote him “ I will not win the Nobel Prize, but my desire is that my students should win a Nobel Prize”.

During one of our weekly lab meetings we had planned a surprise birthday for Mark. When he came into the room he started yelling at all the graduate students and postdoc that he had no published papers for that year. As he was yelling, Ceasar came into the room with birthday cake and candle light on it. Everybody started singing happy birthday and he stopped yelling and joined with a smile.”

**Irene Lisovsky:** Quality Reviewer, Health Canada

Years of contact with Mark: 2007–2017

“The beginning of my graduate career started with an email from Mark that shaped the course of my professional life. In true Mark-fashion, I received an email Who? What? Why here? This was followed by an ‘incorrect recipient, I cannot accept this’ reply and numerous apologetic emails clarifying the confusion and welcoming me to the lab. Mark accepted me to his lab with open arms and, given my move from Vancouver to Montreal, a carrying parental concern for basic living necessities. This welcoming tone resonated from Mark and was echoed by his dedicated team of technicians, administrators & scientists.

What I find impressive about Mark (in addition to his exceptional achievements), and try to implement it in my life, is his acknowledgement and respect of people, along with the will to help. Despite his busy schedule, he always made time to speak with me at conferences. Mark always tried to listen, give advice and support, which was exemplified in mentorship and conversations that lead me to my current position. I feel proud and grateful that he was able to vouch for me. Even though he is not with us today, he is still impacting change and making things happen.”

**Victor Kramer**: Medical Science Liaison, Merck

Years of contact with Mark: 2009–2017

“I’ll never forget my first face-to-face meeting with him. We had been conversing through emails about my previous SIV work when he invited me to meet in Montreal. I poked my german/filipino head into his office and said, “Hi Dr. Wainberg, I’m Victor Kramer.” “*You’re* Victor Kramer?” he said incredulously. I think he was expecting someone more Jewish. By the end of the interview he was printing out articles on Pope Benedict XVI’s stance on HIV (namely that condoms are not the answer in the fight against the epidemic, rather a responsible and moral attitude toward sex was the solution) and jokingly asking how I felt about it as a Catholic. I remember thinking to myself that the next phase of my life was going to be an interesting adventure as a Ph.D. student under Dr. Mark Wainberg. It’s a small story that illustrates some of the facets we were all fortunate enough to experience; scientist, activist, and religious dedication. He was a unique figure throughout the world and I am beyond proud to say that I became who I am today because of my time in his lab. May he rest in peace.” 
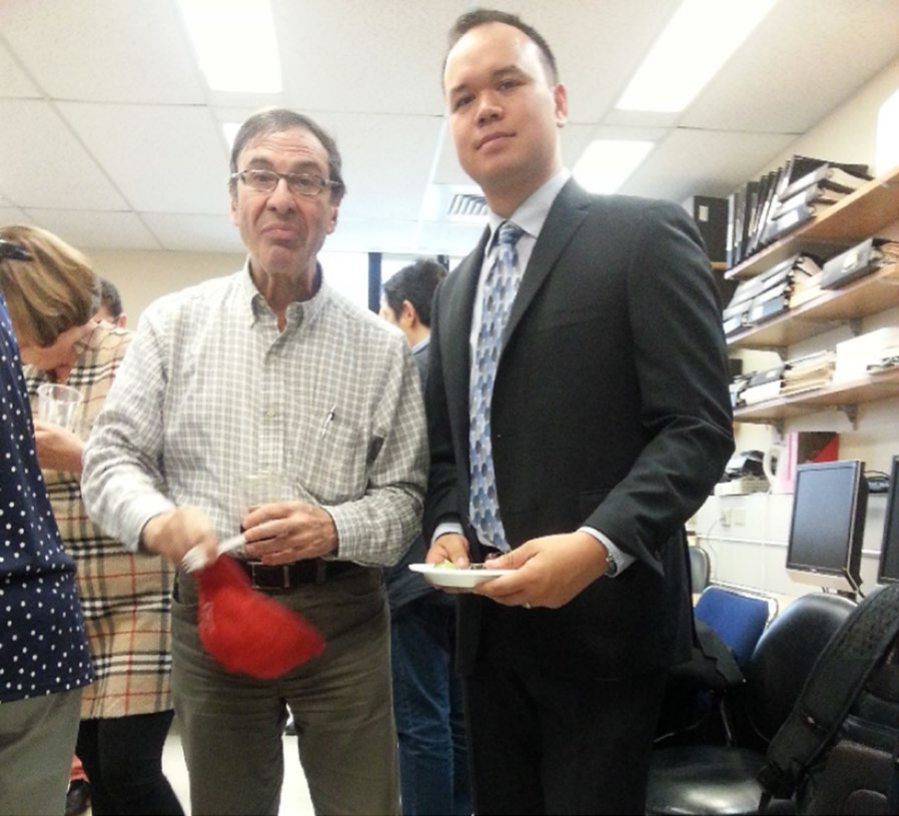


**Éric Cohen:** Professor, Institut de recherches cliniques de Montréal (IRCM)

Years of contact with Mark: 1990–2017

“My first contact with Mark was in the early 1980s as a young graduate student taking my first graduate virology course. I did a presentation in Mark’s course on retroviruses and insertional activation of proto-oncogenes. This presentation as well as the following discussions with Mark had a profound influence on my future scientific interests on retroviruses. Mark inspired me to pursue work on HIV and provided me with encouragement and support while I was a postdoctoral fellow in the Haseltine Lab in the late eighties. I will never forget how our relationship evolved and how we became overtime close colleague and collaborators, participating together in the establishment of CIHR teams, creating what is now the FRQS AIDS Network and organizing HIV conferences. I can still remember that night at the Montreal Concert hall where he informed me with great excitement during the intermission that 3TC-selected mutant of HIV-1 RT displayed enhanced fidelity, a breakthrough that was ultimately published in Science in 1996. I always loved Mark for his optimism, his charism and his friendship. Mark was not only a great scientist but a fantastic human being who made you feel good. He will be greatly missed.”

**Susan Schader:** Co-Principal Investigator, Southern Research

Years of contact with Mark: 2006–2017

“If I am able to see clearly, it is because Mark allowed me to stand on his shoulders”
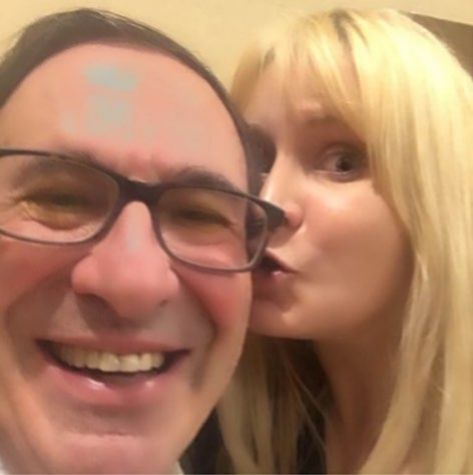


**Agnès Depatureaux:** Residency in Microbiology, Université de Montréal

Years of contact with Mark: 2012–2015

“Dear Mark, thank you for giving me the opportunity to work in your lab. It was a great experience for science and human relationship. I still think you deserve the Nobel Prize…even posthumously. Go on with distilling your tremendous energy in HIV research and wherever you are help us to find the Cure!”

**Chen Liang:** Professor, Lady Davis Institute, McGill University

Years of contact with Mark: 1995–2017

“I joined Mark’s lab as a postdoc in 1995, and I did not know at that time that I would work close to him in the same institute for the upcoming 21 years, until the day he left us all behind. Thinking back, I realize how fortunate I have been to work so closely to such a great mind for so many years, able to observe him, learn from him, and grow into what I am now. Among so many values that Mark has taught me and all his trainees, not just by his words but more by what he has done, is his hard work, his dedication to what he believed in, his courage to fight for what is right, his caring of those who need his help, and most of all, his humbleness as a man who has achieved so much both in science and in humanity. While mourning the sad loss of Mark, which just happened too soon, we also celebrate what a remarkable man he has been, what a splendid and legendary life he has lived, and what he has done to make this world a better place to live.”

**Michel Ntemgwa:** Senior Assessment Officer, Health Canada

Years of contact with Mark: 2004–2009

“In 2004, shortly after completing my Master’s degree in Belgium, Canada was my next destination. One week upon arrival, Dr. Wainberg accepted me with open arms into his lab as technician in Quebec’s Provincial HIV Genotyping Program and later as a Ph.D. Student. Mark was thus my Ph.D. supervisor, mentor and spring board for who I am today. Mark always reached out whenever in Ottawa. Just last year, his former grad students (Hugues Loemba, Sean Li, and myself) hung out with him for dinner, sharing stories and having fun. I was privileged to have attended his 60th birthday Symposium and his 70th birthday surprise event.

My family was blessed with three sons during the time I worked for Mark (2004–2009). During my Ph.D. defence Mark said, “Michel was not only a very productive student but a highly reproductive student”. I wrote to Mark about what my son said when CBC interviewed him upon Health Canada’s approval of Truvada: “Daddy that was your ‘teacher’ in Montreal!”. Mark replied: “Thanks so much but tell Dylan not to watch so much TV. I hope to see you again soon” Thank you for everything Mark. Your contribution towards HIV/AIDS research shall forever be remembered.”

**Jorge Martinez-Cajas:** Assistant Professor of Medicine, Queen’s University

Years of contact with Mark: 2006–2009

“I arrived at Mark’s lab after having completed my fellowship in Infectious Diseases in Detroit. Soon, we started to work on HIV culture in cell lines and PBMCs to investigate drug resistance mutations emerging in different HIV subtypes. He also encouraged me to work on synthesizing clinical data on variations of resistance in different HIV subtypes, and fostered collaboration with fellow post-docs, on various projects. For a clinician like me, the experience of working with Mark was unique, since his lab was involved in many aspects of the HIV field other than pure bench research. Our team of students, post-docs and technicians were people from every continent in the world, and while doing cutting-edge bench research, we also had the chance to do multidisciplinary research and contribute to global educational initiatives such as the HIV [e] education program or the evaluation of impact of HIV education for journalists. Mark enthusiastically supported our collaborative efforts with Colombian partners and traveled twice to Cali, Colombia to teach on HIV resistance to Colombian doctors, researchers and nurses. Mark was a LEADER (with capital case), a mentor who helped many of us discover our full potential as contributors to a better future.”

**Bonnie Spira:** Manager of HIV Laboratories, McGill AIDS CENTRE, Jewish General Hospital

Years of contact with Mark: 1981–2017

“There are no possible words to describe the tremendous sense of loss being felt by Team Wainberg at the McGill AIDS Centre. I have worked with him for 35 years as his manager of HIV Laboratories in Montreal. Early on, as he rose up the ladder to success, he happily and willingly pulled many colleagues along. He took so much pride in the success of his students. He had a passion for science which was only eclipsed by his kindness and compassion and generosity. He was a true leader in the politics of the field. His smile, joie de vivre, and enthusiasm was so infectious that he really made the world a better place. His loss is so tragic. He will be greatly missed.”

**Elana Cherry:** Chief, Clinical Evaluation Division, Autoimmune and Endocrinology, Health Canada, Biologics and Genetic Therapies Directorate

Years of contact with Mark: 1994–2000

“Mark was my post-graduate supervisor. I initially started as an MSc student, but Mark quickly encouraged me to switch to seek a Ph.D. I really appreciated Mark’s supervisory style which was to provide guidance, advice, encouragement, and tools, and then to let us each figure it out.

What I most remember, aside from his walking super-quickly down the hall while almost yelling information intended for someone, was his absolute kindness from a deep place inside him. He was a real person. He loved the work because he loved the work, plain and simple. It was great when he had an idea and he would present it to you for consideration. While he was the boss, the lab felt more like a big collaboration. It was a wonderful learning experience. In my first job interview after graduating, I was asked who I admire in the world. I quickly replied “Dr. Wainberg” because he forged his own path in life and was successful due to simple hard work and determination. For that alone, he has always been a role model to me.”

**Estrella Moyal:** Secretary to Dr. Wainberg

Years of contact with Mark: 1998–2017

“It was when I just started working for him as a secretary. I was intimidated by his stature and doing my best to fit into what was required in my work. Dr. Wainberg travelled a lot. But, the work had to get done at the lab. He asked me if I would mind coming to work on a Sunday. I accepted. I was a bit anxious. On that Sunday morning, I went to his office to let him know that I was in. The first thing he said to me was “Estrella would you like me to make you a coffee”. From that moment on I realized how lucky I was to work for him.

**Anthony Fauci:** Director, National Institute of Allergy and Infectious Diseases, National Institutes of Health Years of contact with Mark: 1984–2017

“Mark was an extraordinary colleague. He generated excitement and “fun” in his own work and in that of his collaborators. Besides being a top-quality scientist, he was one of the most likable people in our field. He had an endearing way of making fun of himself, yet he was profoundly serious when it came to issues such as human rights and the need for greater government support of AIDS research. It is tough to imagine an International AIDS Society meeting without Mark being there.”

**Bonnie Mathieson**: Health Science Administrator for Special Projects NIH, Office of the Director, Office of AIDS Research

Years of contact with Mark: 20+

“Mark first engaged me in discussions of why 3TC escape variants of HIV would make great vaccine candidates because they would be crippled viruses that would not be able to replicate well enough to cause disease. As it became evident that escape variants were still replicating and pathogenic, he modified the premise to argue that they would still make good inserts in vectors. This occurred during the reviews of the original CANVAC which engaged many of the major immunologists and HIV virologists in Canada in the 1990s. Throughout the years, we met at various meetings and discussed the urgency of doing whatever was possible to treat people until we got a successful HIV vaccine. For the past 10–12 years, I was fortunate to join with him and others and the Virology Education group to participate in organizing and conducting the meetings on HIV transmission. Mark was ever ready to urge us to debate controversial topics at the workshops and engage colleagues able to take up opposing views. I will miss his unflagging energy, undeniable excitement for new data, flexibility to grasp new ideas and support new investigators, and willingness to commit himself for the good of others.”

**Jonathan Karn:** Reinberger Professor and Chairman Department of Molecular Biology and Microbiology, Case Western Reserve University School of Medicine

Years of contact with Mark: 1992–2017

“I first met Mark at Brocket Hall in Hatfield, Hertfordshire, England in 1992. The Brocket Hall meeting was the most lavish occasion any of us had ever experienced and has probably never been equaled. We spent a week living in baronial splendor and dining at a table once used by William Lamb, 2nd Viscount Melbourne, who was Queen Victoria’s first Prime Minister from 1835–41. The talks were held in a converted salon and when the time came for Mark to speak, he pulled back the screen to reveal a Van Dyke painting and declared, “Nothing we say here is going to last as long as this or be as valuable.” That was typical of Mark, who loved his science but recognized there were many other important things in life. Mark was a member of our CFAR’s scientific advisory board, and I especially valued his perceptive and politically astute advice. Over the last 2 years we both served on the NCI Board of Scientific Counselors. He would always introduce himself at these meetings by saying his ambition was to Cure HIV before I did, but unfortunately, for all of us, he did not get the chance.”

**Michael Lederman:** Professor of Medicine, Case Western Reserve University

Years of contact with Mark: 1992–2017

“I remember Mark at the podium at a large scientific conference. I can’t remember which one it was but I know it wasn’t the Durban IAS meeting that he chaired. But the issue at hand was the global disparities in AIDS care. Mark stood at the lectern and demanded that each of us advocate for correcting these disparities and work directly to end them. His passion was intense, inflammatory and physically compelling. I think that the existence of injustice was offensive to his soul and demanded resolution. He was my hero.”

**Karidia Diallo:** Health Scientist, Centres for Disease Control and Prevention

Years of contact with Mark:

“My interaction with Mark was the one between a mentor and a mentee. I had all my interactions with him in French, except during my exams. One thing I will never forget is his humility. One day, (May 1st 2001), Mark lost his temper because the department had scheduled my Ph.D. exam with no previous consultation with him. He started yelling as if it was my fault but I just left and went home. Mark later realized he was yelling at the wrong person, looked everywhere, and couldn’t find me. The next day I found a letter on my desk where he was asking for forgiveness. This was a full page written by my mentor, asking me to forgive him! I was very touched by his gesture. I still have that letter today. I said to myself, if Mark can apologize to me, his student, I should never forget this and always apologize when I am wrong, no matter what it is. Merci Dr. Wainberg. You have been silenced, but you’ll never be forgotten!”

**Frank Maldarelli:** Principal Investigator, NCI DRP NIH

Years of contact with Mark: 2000–2017

“Mark was ebullient, with an unrestrainable fervor for the great passions in his life: his family, his country, and his work come immediately to mind. Catching up with Mark at a meeting meant hearing about all three. He dearly loved his Quebec roots: on his last visit to NIH in Bethesda he showed several slides that were entirely in French. He tried to convince the audience, which had nearly filled the largest NIH auditorium, that there was a legal statute that he had to show at least one slide in French or he would not be allowed to return to Quebec. Of course, had there been such a law, it would have been passed at Mark’s insistence. The lecture itself was masterful, comprehensive, erudite, amusing and provocative; in short, it was just like him. Au revoir mon cher ami, tu me manqueras.”

**Michael Miller:** Senior Director of Clinical Virology, Gilead Sciences

Years of Contact with Mark: 1997–2017

“As a young scientist in 1999, I co-published resistance selection experiments with Mark, which first established the K65R mutation as a signature mutation for Tenofovir. Subsequently, we enjoyed each other’s company at every scientific conference on HIV. We had healthy debates on all matters of HIV resistance, including an on-going question as to whether a K65R plus M184 V mutant would ever transmit. Mark’s dedication to the field was remarkable, doing so much more than most scientists. He forced us all to think both scientifically as well as politically. We will all miss his candidness and community service.”

**Andrew Mouland:** Professor, Lady Davis Institute, McGill University

Years of contact with Mark: 2000–2017

“I first met Mark when he had the good sense to hire me back in 2000. I think he liked what I was researching, but he also accepted to share his lab with me, as well as with a few other fledgling investigators. His staff whizzed around me, and it seemed like every week something was being published and another project pursued, and many might still remember the large number of onerous radioactive RT gels. As he became one of the most prolific scientists in Canada, he seemed to orchestrate this with ease with the talented people around him. Some of his secrets I gleaned but many others I never learned. The one that stuck out, but granted it takes a certain personality, was advocacy, by allowing our voices to be heard with authority and appeal. Another such secret is to prioritize the desires, ideas and well-being of his staff and trainees. I never, never had a negative interaction with Mark, but I felt the weight of his authority from six (when he was Director) and more recently, from three floors down. Adieu.”

**Peter Quashie:** Banting Postdoctoral Fellow, University of Toronto

Years of contact with Mark: 2010–2015

“I remember when Mark asked us to figure out a way to recognize integrated HIV DNA in the genome and cut it out. We snickered at him and thought he was crazy…

Mark was my dream supervisor; he was strict, non-controlling, about the science and importantly, about the clinical impact and about productivity. He let you work at your pace but his long leash could get short-instantly! His charisma and passion for the research energized and motivated us to an extent that I only recognized in absentia. His constant pressure of “So when do you think you can wrap this up?” was always followed by a smile while you stammered with timeline projections. He was always quick with a quip or quasi-inappropriate but honest joke. I enjoyed the occasional Friday whiskies. As controversial as his views were, I am in awe of the fact that in all my time in the lab, I do not recall him being wrong.”

**David Dalmau:** Head, HIV/AIDS Unit, Director Research Foundation Mutua Terrassa, Mutua Terrassa University Hospital, University of Barcelona

Years of contact with Mark: 1994–1995, 2002–2008

“While doing my postdoctoral fellowship in Montréal, I met Mark several times at the Jewish General Hospital (general sessions, Kosher hospital restaurant, HIV meetings,…). I also met him frequently at the Canadian HIV Trials meetings. After my return to Barcelona, I met him several times at meetings and specifically at the annual International Workshop on HIV Drug Resistance and Treatment Strategies. He always laughed when I called him Mark184Wainberg.… We’ll miss you, Mark!”

**Siddappa Byrareddy:** Associate Professor, University of Nebraska Medical Centre

Years of Contact with Mark: 2008–2017

“I had great interactions with Mark several times. We initiated collaboration some time ago to test new antiretrovirals (from a study in Mark’s laboratory) in macaque models. Unfortunately, due to funding issues this project was not pursued. I had great interactions with Mark and his students and he asked me several times to come to Canada and work/set up my laboratory. We always exchanged ideas and we recently had great time in Mexico during HIV DART meeting where Mark delivered a fantastic talk. He was a good colleague, a great friend and a mentor. We miss you Mark.”

**Jan Balzarini:** Emeritus Professor, Rega Institute for Medical Research, KU Leuven, Belgium

Years of contact with Mark: 1995–2017

“Mark had a unique combination of a number of qualities: he had a generous, honest, straightforward and social personality; he was a very pleasant company; he was a driven, enthusiastic and ambitious scientist with a very broad, open and sharp scientific view; he was very committed to the society and contributed so much to the field of HIV therapy; he was a good, generous and loyal colleague and friend. In summary, Mark was a person whom everybody would dream of to have closely around! I have an endless appreciation for him! He will be missed by so many!”

**Helen Rees:** Executive Director, Wits RHI, University of Witwatersrand, South Africa

Years of contact with Mark: 1995–2017

“The tragic news of Dr. Mark Wainberg’s death has greatly saddened all of us who knew him. Mark and I worked together as the co-chairs of the first International Microbicide Conference in Washington DC in 2000. Mark was an outstanding scientist who in the course of his career made scientific breakthroughs, while also being a vocal supporter of the human rights of those living with HIV. Mark was also a warm human being with a ready smile and sense of humour, someone who it was always a pleasure to see and to talk to in the many shared meetings that we attended over the years. The field has lost a great champion and the world has lost a special person. Our sympathies go out to Mark’s family.”

**Bechan Sharma:** Professor and Head, Department of Biochemistry University of Allahabad, India

Years of contact with Mark: 2011–2017

“It was October 2011 when I got in direct contact with Prof. Mark Wainberg when I was working as a visiting scientist with Prof. Stefano Sarafianos at the University of Missouri Columbia. Dr. Wainberg was working with Dr. Sarafianos on different projects related to HIV-1 pathogenesis and anti HIV-1 drug design and development. I feel myself fortunate to have had a productive interaction with such a learned scientist in the area of molecular biology of human retrovirology. I found him to be a very humble and modest person with a jubilant mood with encouraging attitude to everyone working in this area of research. The world has lost a great scientist who had dedicated his life in producing wonderful and innovative information in solving the puzzles of biology and pathogenesis of retroviruses.”

**Wendy Wobeser:** Associate Professor, Queen’s University

Years of contact with Mark: 2006–2016

“I received unofficial mentorship with Mark over the years. I believe both our first and our last meetings together were in Glasgow at the EACS meeting probably first about 2006 and last in October of 2016. Back in 2006 we had a conversation about doing a population based phylogenetic analysis of HIV - something yet to come to fruition. Last year the conversation became more personal as Professor Wainberg had suffered a catastrophic failure of a dental crown while in Glasgow of one of his front teeth leaving a jovial and toothless grin. Later was most excited to report that he just happened upon a local Glaswegian dentist who insisted on repairing the unfortunate mishap, something which was done for free. Between the first and last meeting Dr. Wainberg came to Kingston, Ontario to speak to our small group about 3TC resistance which was delivered in our famous waiting room on the 7th floor of the Connell wing, overlooking Lake Ontario. While Drs. Wainberg and Ford enjoyed some light hearted intellectual sparring I started to wonder, based on what we had just heard, whether we should promote resistance to 3TC in light of a potential beneficial effect. Despite his own observations, his concern remained the health of a patient even if that went against his own research position.”

**Michel Alary:** Director, Population Health Research CHU de Québec, Université Laval

Years of contact with Mark: 1991–2017

“I first met Mark at the founding conference of the Canadian Association for HIV Research in 1991. It was for me the first opportunity of one-to-one interaction with Mark as he decided that he would convince me to join the first board of the association as the representative of the epidemiology track. As for most things he undertook in life, he managed to convince me, and then I remained on this board for 10 years, including 2 years as the president, including being the organizer of the 1998 CAHR Conference. Mark deeply influenced my career, especially through his involvement at the global level, an example that I always tried to follow. He regularly supported me by providing letters of reference to different organizations when I applied to host international conferences or when colleagues put forward my name for awards and recognitions. Mark was always there to support me despite his extremely busy schedule. We always had fun together when meeting by chance in airports between two flights, which were the main opportunities we had to meet outside the work context. I last met him at the Montreal CAHR Conference last month, where he was so joyful. I will miss him deeply.” 
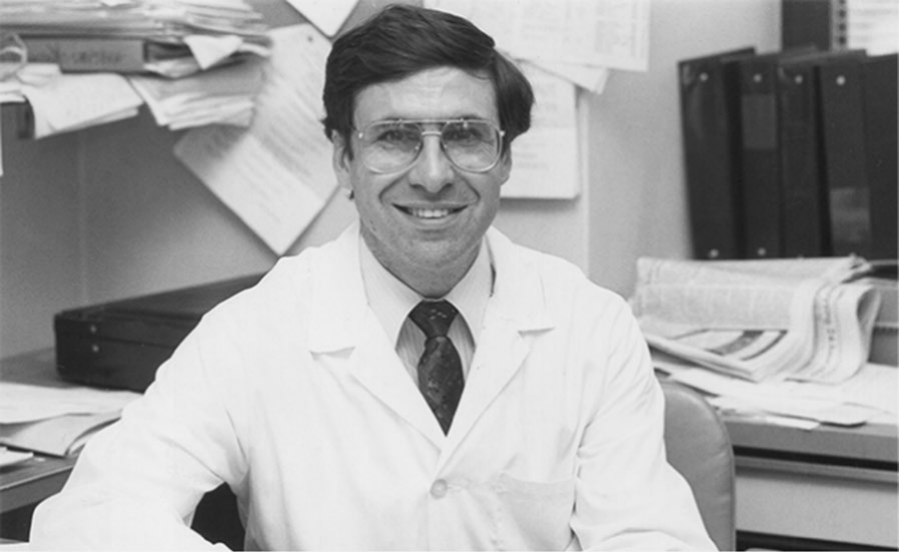


**Linda-Gail Bekker**: President, International AIDS Society

Years of contact with Mark: 2000–2017

“Memories abound around the AIDS conference: Durban 2000 but my memory most recently of Mark was at the Durban AIDS Conference 2016, when his front tooth implant had become dislodged eating an apple! He was so funny and grounded about it- not at all self conscious and kept putting it in and taking it out—so like Mark- famous and smart but grounded enough to be wonderfully funny and quirky about it! This is way beyond the great stories and photos of his family (of whom he was immensely proud)”

**Esteban Martinez:** Senior Consultant & Associate Professor of Medicine, Hospital Clínic & University of Barcelona

Years of contact with Mark: 2003–2017

“I have known Mark for almost 15 years. He hosted me at a meeting in Montreal in 2003. We have met several times along the years when we have participated in different meetings and shared time and topics. We have more recently (last two years) collaborated on several projects regarding resistance to dolutegravir monotherapy.”

**James Whitney**: Assistant Professor, Harvard

Years of contact with Mark: 1999–2003

“Great person, Mentor and friend, will be greatly missed.”

**Anne Gatignol:** Professor and senior investigator, McGill University and Lady Davis Institute for Medical Research

Years of contact with Mark: 1998–2017

“Although he was the head of the International AIDS Society at that time and very busy, he helped me with my recruitment and transition to the Lady Davis Institute. Throughout the years, Mark has been a great colleague, always very enthusiastic about HIV research. He cared very much about the translation of his work to patients everywhere in the world. When I arrived at the LDI, I worked on the same floor and he was very busy interacting with journalists contesting that HIV was the cause of AIDS. In addition to his work, he has been a great advocate to ensure that all patients receive the best treatment. It is in large part because of him that companies accepted that generic drugs were made available for developing countries before the expiration of their patent rights. It is because of him that the access to HIV treatment in the world has now reached more than 50%. He deserves that we continue his work and honour his accomplishments.”

**Ken Rosenthal:** Prof. Dept. Pathology & Molecular Medicine, McMaster University

“Like Mark, I became interested in AIDS even before the virus was isolated and identified. It was clear even then that AIDS was due to a virus and most likely a retrovirus. Over the years, I’ve had a lifetime of interactions with Mark Wainberg. In a nutshell, Mark was like David in the battle against Goliath, whether he was responding to HIV denialist’s like Peter Duesberg or the President of South Africa. Mark always stepped up to the plate and told it as he saw it. He had great strength & conviction. When I had the good fortune of serving as President of the Canadian Association for HIV/AIDS Research (CAHR), I thought it was time to recognize and establish a living tribute to Mark for his accomplishments in fighting HIV over the years and established the annual Wainberg lecture. I also established the Red Ribbon Award to recognize HIV-affected individuals who contributed to the AIDS fight. I’m pleased that Mark, while alive, was recognized annually by his colleagues & peers and I am very sorry to lose such a powerhouse in the fight against HIV/AIDS.”

**Mauro Scehchter:** Professor of Infectious Diseases, Projeto Praça Onze, Universidade Federal do Rio de Janeiro

Years of contact with Mark: 1999–2017

“My warmest and fondest memories are of Mark telling me Jewish jokes at countless late night conversations at bars in hotels around the world. He always had a new one to tell. At those moments, he showed some of what made him such a top scientist and activist and his capacity to laugh about ourselves.”

**Shirin Heidari:** Executive Director & Editor in Chief Reproductive Health Matters

Years of contact with Mark: 2007–2015

“I remember Mark standing at the closing of the AIDS2006 conference in Toronto: “Shame on you, Mr. Prime Minister!”, referring to Mr. Harper, the Canadian Prime Minister at the time who was conspicuously absent. It was wonderful to see a world-known scientist being such a vocal activist. Later in 2007, I came to join the International AIDS Society, and was delighted to be working closely with Mark. During his IAS presidency, Mark had founded the Journal of the International AIDS Society. His vision was as a platform to support dissemination of HIV research by younger investigators, in particular those from low- and middle-income country. As the executive editor of the journal, I came to work very closely with Mark for seven years. Together with the other editors-in-chief, we developed the journal and organized capacity building workshops, and defended the journal in face of financial constrained that threatened its existence. During these years, I came to know him; not only a dedicated and committed scientist, but a wonderful human being with a kind heart. I will always remember him with his broad happy smile. Mark, you will be truly missed! Rest in peace, and rest assured that your spirit will live forever.”

**Wamarou Traore:** Head of Health Department & Global Fund National Coordinator, National Permanent fighting against AIDS Council

Years of contact with Mark: 2002–2006

“Many times he was chairperson of our working technical groups studying mothers and children infected by HIV in four countries of West Africa Burkina: Faso, Ivory cost, Mali and Senegal, projects funded by Bristol Myers Squibb. Mark, Rest in peace !!!”

**Veronica Miller:** School of Public Health, UC Berkeley

Year of contact with Mark: 1996–2017

“I remember Mark for his enthusiasm – it never let up or tired. He was one-of-a-kind. His contributions to the field as a scientist and as a mensch are legendary.”

**Josee Brisebois:** Director Medical Affairs, Gilead Sciences Canada

Year of contact with Mark: 1996–2017

“From training in his lab (my 1st P3 experience), to my thesis defense and our subsequent and numerous collaborations in the context of pharma antiretroviral development, Mark has had a profound impact on my career and those of so many of my colleagues. His passion, his compassion, his dedication, his sense of humour. Merci pour tout Mark!”

**Jonathan Schapiro**: Director HIV/AIDS, National Hemophilia Center, Israel

Years of contact with Mark: 25

“Mark was an observant and thoughtful Jew. But unlike many these days who use their religion as an excuse for racism or violence, Mark always found a way of channeling his Jewish faith to humanitarian causes. Here is an email I received from my good friend Mark in early February 2016.

Hi Jonathan,

It was good to see you at the conference. Attached is the article from the Jerusalem Post on the Torah donation as well as a commentary by the current Rabbi of Kehal Yushurun Synagogue in Manhattan. For me this was way more important than another paper on Dolutegravir, even though I hope and believe that our Dolutegravir hypothesis will be accepted before long by all. I may decide to soon pack it in, make Aliyah, and mostly lie on the beach in Herzlya.

Regards,

Mark”

**Romina Quercia:** Global Director of Clinical Virology Development, ViiV Healthcare

Years of contact with Mark: 2005–2017

He is and was the “chef” with the curiosity of a child and spirit of a young Ph.D. student. Mark was ageless and timeless. I had the opportunity to share Ad Boards and discuss projects with him and he was teaching “by doing” all the time. He was always the first to send a draft, the first to review, the first to defend an idea. A loyal, kind and a true researcher. A believer. Our last anecdote is very recent…just few weeks ago. We were writing a paper and I asked all the authors to send a draft of their respective sections, the deadline was 3 weeks. Mark sent it 40 mins after I requested it…plus questions, plus comments and suggestions. I still have the draft and I cannot stop reading it. The draft article exemplifies his spirit and strength: straight to the point, provocative and passionate. He was very knowledgeable but his ultimate value was his passion and imagination. As Albert Einstein said “Imagination is more important than Knowledge”. I can define the “Chef” Mark Wainberg as the last “Provocateur of Imagination and Knowledge” in our field.”
